# Effectiveness of two-stage inoculation of *Bacillus subtilis* and *Bacillus megaterium* on nitrogen transformation and microbial community in cow manure composting under low temperatures

**DOI:** 10.1128/spectrum.01878-25

**Published:** 2026-03-12

**Authors:** Zhengjin Zhu, Zhiming Xu, Xiu Zhang, Penghui Sun, Weimiao Ni, Haoduo Bai, Cleophas Achisa Mecha, Ronghua Li, Fusheng Quan

**Affiliations:** 1 College of Veterinary Medicine, Northwest A&F University 718173, Yangling, Shaanxi, China; 2 Laboratory of Gastrointestinal Microbiology, Jiangsu Key Laboratory of Gastrointestinal Nutrition and Animal Health, National Center for International Research on Animal Gut Nutrition, College of Animal Science and Technology, Nanjing Agricultural University 524556, Nanjing, China; 3 North Minzu University Ningxia Key Laboratory for the Development and Application of Microbial Resources in Extreme Environments, Yinchuan, China; 4 School of Natural Resources and Environment, NWAFU 12469, Yangling, Shaanxi, China; 5 The Department of Environmental Science, The University of Arizona (UA) 8041, Tucson, Arizona, USA; 6 School of Natural Resources & Environment, NWAFU-UA micro-campus, Yangling, China; University of Mississippi, University, Mississippi, USA

**Keywords:** two-stage inoculation, low-temperature composting, psychrophilic microbial consortium agent, nitrogen transformation, microbial community structure

## Abstract

This study examined the effects of two-stage inoculation with psychrophilic New-Cold adapted Microbial Consortium Agent (NCMCA) on nitrogen transformation and microbial community dynamics during composting of cow manure under low ambient air temperatures (1.2°C–12.6°C). The composting efficiency under low temperatures was markedly enhanced compared with the control. After the NCMCA was inoculated, the duration of the thermophilic stage was doubled to 6 days, and the moisture content reduced by 5.99% (*P* = 0.006). NCMCA inoculation enhanced nitrogen retention, reducing cumulative NH_3_ emissions by 11.82% (*P* = 0.001) and increasing total Kjeldahl nitrogen and NO₃⁻-N content by 9.58% (*P* = 0.01) and 42.03% (*P* = 0.03), respectively. Microbial community succession indicated enhanced Proteobacteria and Gemmatimonadota, while reducing Chloroflexi, Actinobacteriota, and Bacteroidota at the phylum level. At the genus level, NCMCA inoculation increased the proportion of *Luteimonas*, *Hydrogenophaga*, and *Devosia* and decreased the relative abundance of *Candidatus_Chloroploca*, *Ruminofilibacter*, and *Thermopolyspora*. The two-stage inoculation of NCMCA produced superior-quality compost with enhanced nitrogen retention and significantly increased bacterial diversity compared to that of traditional composting.

**IMPORTANCE:**

This study addresses key challenges in low-temperature composting and provides solutions for cold-region agricultural sustainability and waste management. Using two-stage inoculation of a psychrophilic microbial consortium agent (New-Cold adapted Microbial Consortium Agent [NCMCA], *Bacillus subtilis*, and *Bacillus megaterium*), during cow manure composting under 1.2°C–12.6°C provides significant performance improvement. NCMCA doubles thermophilic phase duration, elevates peak temperatures, and accelerates decomposition, overcoming cold-induced microbial inhibition. Notably, it reduces NH₃ emissions by 11.82%, increases total nitrogen and nitrate levels, boosts compost quality, and mitigates environmental risks. By revealing how the NCMCA modulates microbial communities to promote nutrient cycling, this cost-effective strategy advances sustainable waste treatment in cold climates, supporting eco-friendly agriculture.

## INTRODUCTION

Cow manure, a major byproduct of the livestock industry, poses multiple environmental and management challenges. Traditional disposal methods, such as direct stacking or land application, consume substantial land resources and pose significant environmental risks, including odor emissions, spread of pathogens, and potential heavy metal pollution; all these threaten soil health and food safety (1). Although livestock and poultry manure are a valuable bioenergy source (2), their utilization is hindered by improper disposal, releasing significant amounts of nitrous oxide (N_2_O), methane (CH_4_), and ammonia (NH_3_) (3). Composting involves piling up livestock and poultry manure and crop straws after harvest, thus transforming the organic matter (OM) into organic fertilizer, eliminating pathogens, reducing the volume of organic waste, and enhancing the nutrient cycle (4). However, traditional composting results in nitrogen loss due to single material conditions (5).

Additives, such as biochar, zeolite, organic acid, and iron salts, have been used to mitigate nitrogen loss, facilitate organics conversion, and safeguard the environment (4, 6, 7). However, aerobic composting is essentially a biochemical reaction process driven by various microbial communities (8), and as such, extending the thermophilic phase, promoting OM decomposition, and reducing the emission of greenhouse gases and other pollutants have gained more interest. Studies on microbial agents directly inoculated into compost substrate at the initial stage or at the cooling stage (3, 9), including thermophilic microbial agents such as *Bacillus* (10) and thermophilic actinomyces (11); cellulose-decomposing bacteria such as *Trichoderma* (12), *Aspergillus* (13), and *Xenophilus azovorans* (3); and consortium microbial agent with Fenton-like functions (14), have been reported. However, there are very limited studies on the utilization of cold-adapted microbial agents.

Livestock-producing regions in China (e.g., Xinjiang, Ningxia, Inner Mongolia, and Qinghai) experience relatively low winter temperatures, which inhibit microbial activity because the heat generated is insufficient to initiate composting (15). Psychrophilic microorganisms employ multiple adaptive mechanisms, such as the efficient catalysis of cold-active enzymes (16, 17), maintenance of cell membrane fluidity (18), optimization of metabolic pathways (19), anti-freezing protection (19), and community collaboration (20), to enhance composting by overcoming the limitations imposed by low temperatures on the rate of chemical reactions and biological activity. For instance, *Pseudomonas mendocina* X49 demonstrated high simultaneous nitrification and denitrification under heterotrophic aerobic conditions (21).

Thus, the introduction of psychrophilic microorganisms rapidly initiates and continuously decomposes OM at low temperatures (22). They are more cost-effective and energy-efficient compared to plastic sheeting, insulation, and fire heating, which were traditionally used (23). In addition, the two-stage inoculation of microbial consortium at the initial stage and at the cooling stage elevates the compost temperature and extends the thermophilic period (24), strengthens bacterial networks and activity (25), promotes organic carbon conversion (26, 27), favors nitrogen transformation, reduces nitrogen loss, and improves compost quality (9), by triggering secondary aerobic fermentation (28). We postulate that the use of a psychrophilic microbial consortium agent will ensure effective low-temperature composting, and that two-stage inoculation could facilitate nitrogen transformation and compost stabilization. The present study was designed to evaluate these aspects.

In this study, we isolated two highly efficient psychrophilic strains, namely *Bacillus subtilis* and *Bacillus megaterium,* and formulated NCMCA. *B. subtilis* effectively promotes cellulose degradation and forms spores to survive in adverse conditions (29), while *B. megaterium* increases soil nutrient content and the relative abundance of beneficial soil bacteria and fungi while suppressing pathogenic bacterial genera (30). We applied two-stage inoculation of cow manure composting (days 1 and 28) under cold conditions. The specific objectives were to: (i) assess the effect of NCMCA on composting maturity and stabilization, (ii) evaluate the function of two-stage inoculation of NCMCA on harmful emissions during composting, and (iii) analyze bacterial community abundance and the interaction mechanism between microorganisms and environmental factors.

## MATERIALS AND METHODS

### Materials pretreatment

Fresh cow manure and dry wheat straw were sourced from the Animal Breeding Test Station and the Agricultural Test Farm of Northwest A&F University, China, respectively. Dry wheat straw was milled into powder (less than 2 mm) before use. NCMCA (*B. subtilis:B. megaterium,* 1:1) was prepared in the laboratory by screening cold-adapted microorganisms from winter cow manure and frozen soil. It consisted of *B. subtilis* (optimal growth temperature, 10°C–30°C) and *B. megaterium* (a cold-adapted bacterium with a growth-promoting temperature of 10°C). The inoculation concentration of NCMCA was 1 × 10⁸ CFU/mL. The properties of cow manure and wheat straw are shown in Table 1.

**TABLE 1:** Physicochemical parameters of aerobic composting materials (dry weight basis)^*a*^

Parameter	Cow manure	Wheat straw
Moisture content (%)	68.79 ± 0.38	6.17 ± 0.09
EC (mS/cm)	2.57 ± 0.64	0.87 ± 0.07
pH	8.92 ± 0.16	7.34 ± 0.15
TKN (g/kg)	14.36 ± 0.75	7.83 ± 0.25
TOM (g/kg)	627.26 ± 0.71	928.64 ± 4.72
TOC (g/kg)	372.18 ± 6.22	585.68 ± 6.29
C/N	19.34 ± 0.81	67.56 ± 2.68

^*a*^ EC, electrical conductivity; TKN, total Kjeldahl nitrogen; TOM, total organic matter; TOC, total organic carbon; C/N, carbon/nitrogen ratio. The results are the mean of three replicates ± standard deviation.

### Compost preparation

Cow manure and wheat straw powder were thoroughly mixed with a moisture content (MC) of 65% and a C/N ratio of 30 for the mixture. The compost mixture was divided into two parts: one part without inoculation (replaced by sterile water) and denoted as the control treatment. Because the metabolic rate of microorganisms and the fermentation temperature are low on day 1 (initial stage) and day 28 (cooling stage) of composting, the other part was inoculated with 5% (vol/wt) NCMCA (1 × 10^8^) during these two periods and labeled as the NCMCA treatment (31, 32). The mixtures of both treatments were composted in three separate 100 L aerobic reactors with ventilation devices set up, as described in a previous study (31), for 56 days. The compost was turned once in the first 3 days and once every 7 days thereafter to ensure homogeneity.

### Sample collection and index analysis

**Fig 1:**
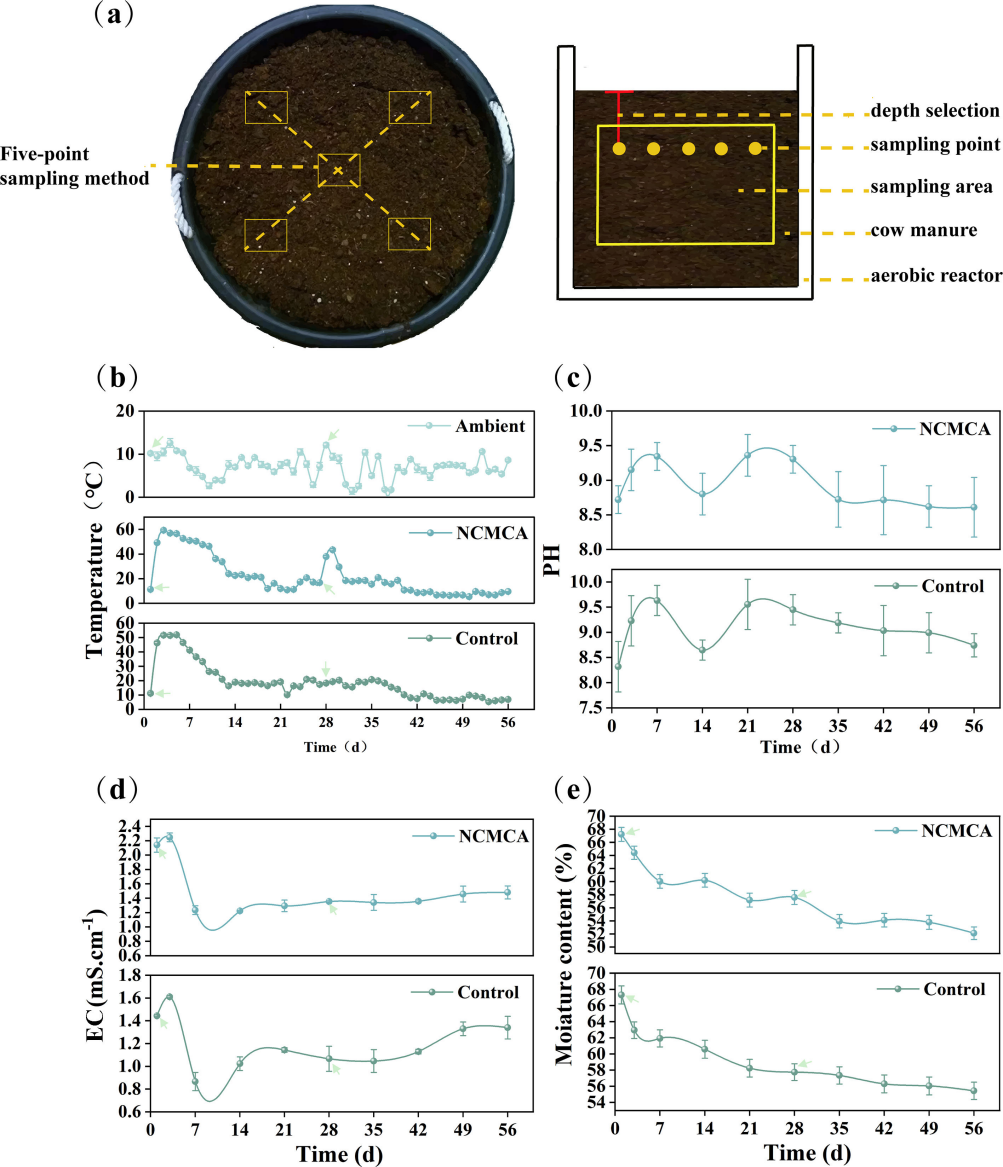
Schematic diagram of the compost sampling area (**a**) and changes in the temperature (**b**), pH (**c**), EC (**d**), and MC (**e**). Note: The arrows represent the inoculation of 5% (vol/wt) NCMCA in cow manure on day 1 and day 28, while the control was inoculated with a similar amount of sterile water.

As shown in Fig. 1a, the standard five-point sampling method was employed (33). The sampling depth exceeded one-tenth of the compost pile thickness, and the sampling depth to the compost thickness ratio was maintained consistent. Every day at 9 am and 4 pm, a stainless-steel shovel was used to expose the designated sampling point, and a thermometer was inserted to record the temperature. This procedure was performed for each of the five sampling points. At the same time, a compost sample (100 g) was collected and shredded using sterilized scissors and kept in aluminum foil paper. The five samples were thoroughly mixed and then divided into three parts. Similar procedures were used for the other two aerobic reactors. One portion was air-dried for total Kjeldahl nitrogen (TKN) analysis; the second portion was used for MC, pH, EC, NH_4_^+^, and NO_3_^-^ analysis; and the third portion was stored at −80°C for bacterial analysis.

Constant temperature drying method at 105°C was used to determine the MC. For pH and EC, samples were mixed with deionized water in a 1:10 ratio (wt/vol) and mixed for 2 h, and measured using a standard pH meter and an electric conductivity meter (8), respectively. Nitrous oxide (N_2_O) was measured once a day for the first 2 weeks and thereafter every 2 days using gas chromatography (Agilent Technologies 7890B Network GC system, America). NH_3_ was collected using a CD-3A gas sampler (Beijing Toyi Instrument Co., LTD., China) at an air flow rate of 0.5 L/min for 5 min, absorbed in 2% boric acid in a washing bottle, and then titrated with 0.05 mol/L H_2_SO_4_ solution (9). This was done once a day for the first 2 weeks and thereafter every 2 days for the remaining period. TKN content was measured following the method described in a previous study (5). For NH_4_^+^ and NO_3_^-^ content, 5 g of the sample was extracted with 1 mol/L KCl (1:20 wt/vol) for 30 min at 160 rpm, and the filtrate was analyzed using a flow analyzer (5). All the tests were done in triplicate, and the average values were recorded.

### High-throughput sequencing

The abundance of the bacterial community in the process of cow manure composting was investigated through 16S rDNA amplification and sequencing. Samples of the compost were obtained on days 0, 7, 28, and 56. The samples from the control were labeled as D0_Control, D7_Control, D28_Control, and D56_Control, while those from the treatment group were labeled as D7_NCMCA, D28_NCMCA, and D56_NCMCA. According to the instructions of Uniagaon Biol-Tech. Co., LTD., China, the microbiome’s total DNA was isolated using the CTAB method. The V3–V4 region was amplified using primers 341F (5′-CCTACGGGNGGCWGCAG-3′) and 805R (5′-GactachvGGGTATctaATCC-3′). Ultrapure water was used as a negative control. The PCR products were purified with AMPure XT beads (Beckman Coulter Genomics, Danvers, MA, USA) and quantified with Qubit (Invitrogen, USA). The PCR reaction system and procedure are shown in Table 2.

**TABLE 2:** PCR amplification reaction conditions

Procedure	Temperature (°C)	Time	Cycle number
Predegeneration	98°C	30 s	35 cycles
Denaturation	98°C	10 s	
Annealing	54°C	30 s	
Extension	72°C	45 s	
Final extension	72°C	10 min	
Terminal extension	4°C	∞	

### Quantification of the *nirS*, *nirK*, *nosZ,* and *amoA* genes

Each DNA sample was subjected to three replicates of real-time fluorescence quantitative polymerase chain reaction (qPCR) for the quantitative analysis of the abundances of *nirS*, *nirk*, *nosZ,* and *amoA*. The primer instruments used were consistent with those previously employed (34). The primer sequences are listed in Table 3.

**TABLE 3:** The primers used for qPCR^*a*^

Primers	Target genes	Nucleotide sequences (5′−3′)	Fragment size (bp)
nirK1-F	*nirK*	ATYGGCGGVCAYGGCGA	164
nirK1-R		GCCTCGATCAGRTTRTGG	
nosZ-F	*nosZ*	CGCTGTTCITCGACAGYCAG	700
nosZ-R		ATGTGCAKIGCRTGGCAGAA	
nirS-F	*nirS*	GTSAACGTSAAGGARACSGG	400
nirS-R		GASTTCGGRTGSGTCTTGA	
amo A-lF	Bacterial *amoA*	GGGGTTTCTACTGGTGGT	490
amo A-2R-KS		CCCCTCKGSAAAGCCTTCTTC	
16S DNA-F	*16S DNA*	ATGGCTGTCGTCAGCT	357
16S DNA-R		ACGGGCGGTGTGTAC	

^*a*^ The amplification programs for quantitative PCR, pre-denaturation (30 s, 95°C), denaturation (10 s, 95°C), annealing (30 s, 60°C), and cycle number = 40. Melt curve stage: 95°C for 15 s, 60°C for 1 min, and 95°C for 1 s.

### Statistical analysis

The data were analyzed using Microsoft Excel 10.0, and structural equation modeling (SEM), as well as statistical significance analysis, was carried out using IBM SPSS Statistics 27 at a level of *P <* 0.05. The figures were drawn using Origin 2025 and GraphPad Prism 9. Adobe Illustrator CC 2018 was used to draw group, summary, and mechanism diagrams.

## RESULTS AND DISCUSSION

### Changes of biochemical parameters during composting

As shown in Fig. 1b, the ambient ranged from 1.2°C to 12.6°C, mostly below 10°C. In the initial phase, the temperature of control and NCMCA treatments increased rapidly to the thermophilic phase (>50°C) on the third day, reaching peak temperatures of 52.2°C (Control) and 59.8°C (NCMCA), respectively. The thermophilic phase lasted for 6 days in the NCMCA treatment and 3 days in the control treatment, indicating that inoculation of NCMCA increased the peak temperature and prolonged the thermophilic period. The trends in both groups were consistent, indicating the effect of the dominant bacteria under low temperatures (32). High temperatures led to the death of most microorganisms, and subsequently, the temperature of compost gradually decreased (9), approaching environmental temperature by day 22, resulting in low microbial activity and metabolism. On day 28, after re-inoculating the NCMCA, the temperature increased to 47.4°C on day 29, while the control showed no significant change, thus confirming the warming effect of NCMCA. Because the easily degradable OM, such as soluble sugars, starch, and organic acids, had been degraded in the early stage (3), the temperature of composting after the second inoculation did not rise to the level of the first inoculation of NCMCA. Three days after the second inoculation (day 31), the temperature of NCMCA treatment decreased to the same level as control and then reduced to the environmental temperature at day 45. Similar findings were reported in swine manure composting incorporating a microbial consortia of *Bacillus licheniformis, Bacillus subtilis,* and *Penicillium chrysogenum* at the cooling stage (9).

Figure 1c shows that at the beginning, the pH value increased from ~8.72 to peak levels on day 7, reaching 9.63 (Control) and 9.34 (NCMCA). Subsequently, the pH value leveled and then reduced to 8.61. The overall trend of pH value changes was not affected by the re-inoculation of 5% NCMCA on day 28, which is in agreement with the literature (35). The pH increase is due to the expenditure of substrates that promoted ammonification and the degradation of small-molecular acids (3, 5). The reduction in pH value might be due to the transformation of nitrogen and other compounds (35).

The EC increased (Fig. 1d) as the decomposition of OM released soluble salts (36). Thereafter, it decreased as fermentation progressed due to the evaporation of ammonia and the deposition of inorganic salts (37). Throughout the process, the EC of NCMCA treatment remained higher than that of the control due to the promotion of OM decomposition by NCMCA, causing an increase in soluble salts. The re-inoculation of 5% NCMCA on day 28 did not alter the overall trend of the EC because of the rapid decomposition of easily degradable organic substances coupled with the “concentration effect” (38). By the end of the composting, the EC values of both groups were less than 4.0 mS/cm, remaining within the safe range (36).

As shown in Fig. 1e, the MC of NCMCA treatment and the control exhibited a similar downward trend, decreasing rapidly during the thermophilic period. The MC dropped from 67.24% to 60.03% (NCMCA treatment) within the first 7 days (10.72% decrease: while it decreased from 67.31% to 62.94% [Control] within 3 days [6.49% decrease]). Subsequently, the MC in the control decreased at a slower pace, while the NCMCA treatment witnessed a rapid decrease due to the accelerated metabolism and elevated temperature resulting from NCMCA inoculation. The MC was 57.58% on day 28; after re-inoculating 5% NCMCA, the MC dropped to 53.93% on day 35 (6.34% decrease); the corresponding MC values for the control were 57.75% on day 28 and 57.34% on day 35 (0.71% decrease). Subsequently, the MC continued to decline slowly up to the end of composting.

### Nitrogen dynamic changes

**Fig 2:**
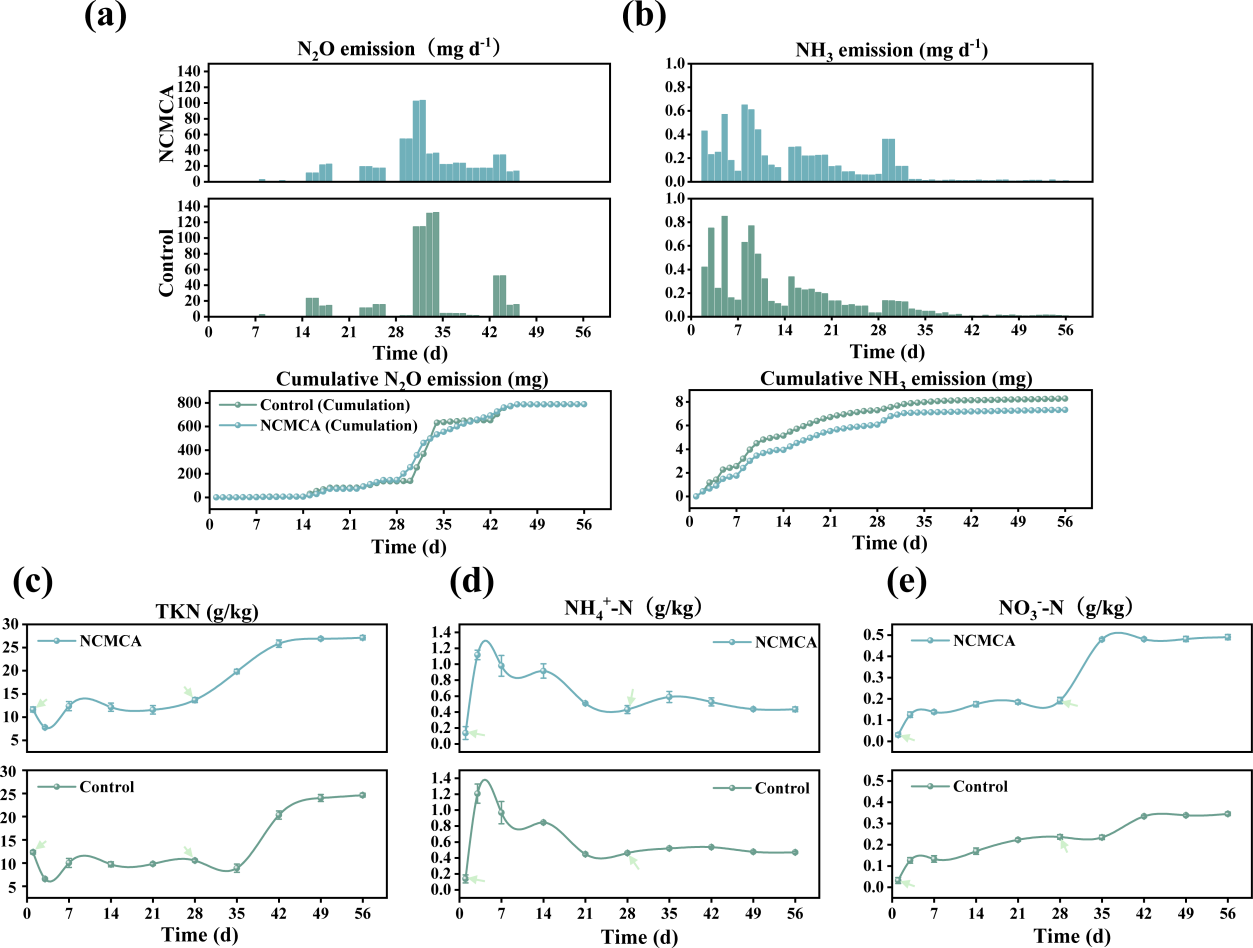
Changes in daily emissions and cumulative emissions of N_2_O (**a**), NH_3_ (**b**), TKN (**c**), NH_4_^+^-N (**d**), and NO_3_^−^-N (**e**) during composting.

The dynamic changes in nitrogen also have a crucial impact on the composting effect and the environment because N_2_O is formed through the nitrification and denitrification processes, and it is a greenhouse gas with a global warming potential of 298 (39), as depicted in Fig. 2a. At the beginning, the emission of N_2_O was extremely low because the high temperature inhibited growth and reproduction of denitrifying bacteria, thereby restricting denitrification (5). After 14 days, as the temperature decreased, the activity of denitrifying bacteria increased, causing the N_2_O content to gradually rise. However, due to the low NO_3_^-^ content (Fig. 2e) and the high pH value (Fig. 1c), the increase in N_2_O emissions was gradual. The peak emission of N_2_O occurred on day 28 after re-inoculation with either 5% NCMCA or sterile water. In the control, the lower temperature (Fig. 1b), higher pH value (Fig. 1c), and increased NO_3_^-^ content (Fig. 2e) led to a sharp increase in N_2_O emissions through nitrification and denitrification of NH_4_^+^and NO_3_^-^ (9). In the NCMCA treatment, the increase in temperature was unfavorable for the metabolism of denitrifying bacteria (5), resulting in a lower N_2_O emission compared to control. The N_2_O was emitted at various intervals, and by the end of composting, the cumulative N_2_O emissions were 788.61 mg and 788.35 mg for NCMCA treatment and control, respectively. Thus, the addition of NCMCA had a negligible effect on the total N_2_O emissions.

Nitrogen loss in aerobic composting is attributed to the release of NH_3_, which reduces the value of agricultural products and threatens environmental hygiene (40). Figure 2b depicts the release of NH_3_ during composting. In the first 10 days under high temperature and pH, the daily release of NH_3_ increased significantly (9). The control reached a peak of 0.85 g on day 5, while NCMCA treatment peaked at 0.65 g on day 8. As the temperature decreased (Fig. 1b), the release of NH_3_ gradually declined (41). After re-inoculating 5% NCMCA on day 28, a temporary resurgence in the daily NH_3_ emission was observed, which could be due to the increase in temperature (Fig. 1b) (9). Two days later, when the temperature returned to normal, the daily NH_3_ emission also decreased to its previous level. The cumulative NH_3_ emission in the NCMCA treatment (7.30 g) was lower than that in the control (8.28 g), representing an 11.86% reduction (31, 32, 34). This implied that the addition of NCMCA inhibited the activity of ammonia-oxidizing bacteria, making NCMCA beneficial for nitrogen retention.

The TKN value reduced from 11.94 to 7.77 g/kg (NCMCA treatment) and from 12.12 to 6.57 g/kg (Control), a 46.67% reduction in the 3 days prior to composting (Fig. 2c). This decline in TKN was due to the substantial release of NH_3_ during the thermophilic phase (5), elevated temperatures, and high pH values, which are not conducive to nitrification (9). From day 3 to day 7, TKN increased, followed by fluctuations within a certain range. After day 28, following the re-inoculation of 5% NCMCA, TKN in the NCMCA treatment increased, while in the control, it initially decreased before resuming an upward trend. The continuous increase in TKN can be attributed to the “concentration effect,” resulting from progressive mineralization of OM. By the end of the composting, TKN levels reached 27.12 g/kg and 24.64 g/kg in the NCMCA and control, respectively, representing increases of 1.32 times and one time. This further demonstrated that the addition of NCMCA is beneficial for nitrogen retention due to reduced NH_3_ release after NCMCA incorporation (Fig. 2b).

The variation in NH_4_^+^-N concentration reflects the transformation of nitrogen forms and the emission of NH_3_ (Fig. 2d). At the beginning, the concentrations of NH_4_^+^-N in the NCMCA and the control treatments were 137 and 138 mg/kg, respectively. The NH_4_^+^-N content in both groups increased rapidly and peaked within 3 days due to rapid mineralization and ammonification of nitrogen-containing compounds that are temperature-dependent (8). As composting advanced, the NH_4_^+^-N concentration decreased due to the conversion of some ammonium ions into free ammonia (Fig. 2b), release into the environment, as well as nitrification-mediated transformation of NH_4_^+^-N into NO_3_^-^-N (Fig. 2e) (5). The re-inoculation of 5% NCMCA on day 28 did not alter the trend of NH_4_^+^-N. Throughout the composting, NH_4_^+^-N levels in the two treatment groups remained relatively similar, and at the end, NH_4_^+^-N concentrations were 435.2 mg/kg (NCMCA) and 470.4 mg/kg (Control), representing increases of 2.18 and 2.41 times, respectively. The lower NH_4_^+^-N concentration indicated that the addition of NCMCA can reduce the NH_4_^+^-N content.

The fluctuation of NO_3_^-^-N content is presented in Fig. 2e. During the initial phase of composting, the concentration of NO_3_^-^-N was relatively low, with values of 30.2 and 31.3 mg/kg for NCMCA and control, respectively. Thereafter, the NO_3_^-^-N concentration in both groups gradually increased. On day 28, after inoculation with 5% NCMCA, there was a significant increase in the NO_3_^-^-N content compared to the control treatment (*P* < 0.05) due to the stimulation of metabolic activity of nitrifying bacteria by NCMCA inoculation, enhancing the alteration from NH_4_^+^ to NO_3_^-^ (9). The NO_3_^-^-N concentration increased 16-fold (NCMCA treatment) and 11-fold (Control), implying that the addition of NCMCA promotes NO_3_^-^-N generation.

Table 4 further indicates that the NCMCA treatment can promote the conversion of nitrogen to stable organic nitrogen, inhibit the denitrification loss of nitrate nitrogen, and reduce ammonia volatilization. Without increasing gaseous nitrogen loss, it significantly increased the residual nitrogen content in compost, resulting in a higher nitrogen recovery efficiency. This confirms the effectiveness of the nitrogen-preserving measures in the NCMCA treatment, in improving nitrogen stability, and in reducing mineralization loss.

**TABLE 4:** Recovery of initial N during composting

Group	Initial total *N* (mg)	Final residual *N* (TKN+NO_3_⁻, mg)	Final residual NH_4_^+^ (mg)	Final emitted *N* (NH_3_+N_2_O, mg)	Final total *N* (mg)	Nitrogen recovery efficiency (%)
Con	247,026	181,640.35	3,415.104	796.914	182,437.26	73.85%
NCMCA	233,404	212,145.6	3,159.552	795.664	212,941.3	91.23%

### Abundance and diversity of bacterial communities

Indices such as Chao1, Observed species, Good’s coverage, Shannon, Simpson, and Pielou’s evenness were employed to evaluate the abundance and diversity of microbial communities in the composting environment (3).

**Fig 3:**
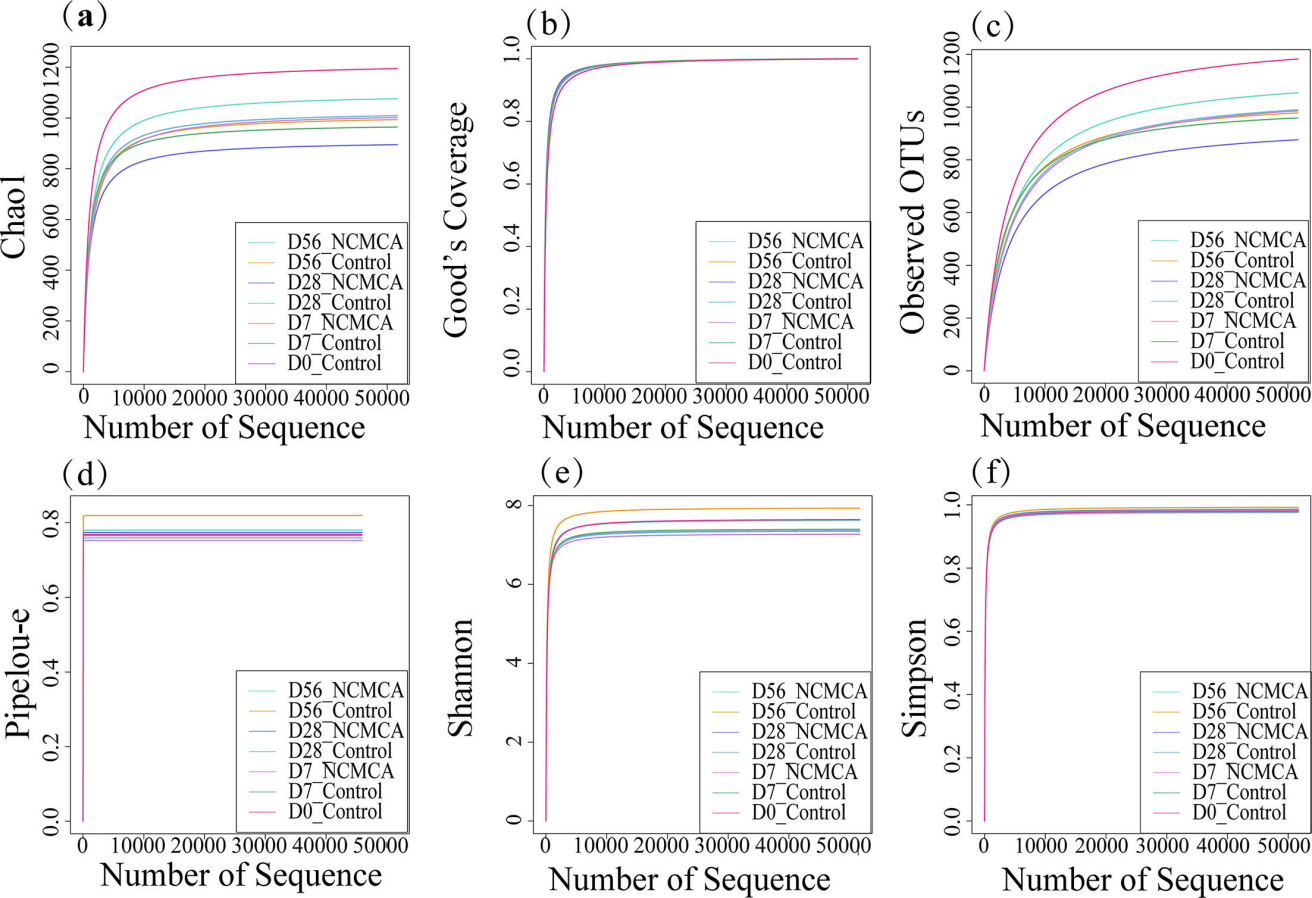
Determination of alpha diversity of bacterial communities. (**a**) Chao1, (**b**) Good’s coverage, (**c**) Observed OTUs, (**d**) Pielou-e, (**e**) Shannon and (**f**) Simpson.

For Chao1 (Fig. 3a), the two treatments displayed opposite trends: the control increased initially (day 28) and then slightly decreased (day 56), while the NCMCA treatment decreased first (day 28) and then increased significantly (day 56). This suggests that the NCMCA treatment enriched many potential species in the late stage and was more sensitive to rare species. Good’s coverage index reflects the degree of microbial coverage, and all sequencing results for this metric equaled 1, accurately representing the true composition of the samples (Fig. 3b). As shown in Fig. 3c, bacterial community abundance was highest during the early composting stage (D0) because the abundance of substrates provided favorable conditions for bacterial growth and reproduction. During the thermophilic phase (D7), abundance decreased as elevated temperatures inhibited the proliferation of certain bacteria, and heat-resistant bacteria became the dominant population, facilitating the decomposition and release of nutrient elements (3). The Observed OTUs at the initial and thermophilic phase were higher than those in the mature phase (Fig. 3c), indicating a substantial difference in the bacterial community at each composting stage (42). In control, richness increased significantly during the cooling stage (D28) and remained at a high level in the late composting stage (D56). In contrast, the abundance of the NCMCA treatment decreased during the cooling stage but increased substantially in the late stage, reflecting the stage-specific nature of the treatment effect. Moreover, the species richness of the NCMCA treatment was higher than those in the control, as expected and consistent with the previous findings (32). Pielou’s evenness (Fig. 3d) and the Shannon index (Fig. 3e), derived from information entropy, indicate greater uncertainty and higher diversity in the bacterial community in composting (7). The values of the Simpson index were close to 1 (Fig. 3f), reflecting high community abundance and uniformity (3).

Chao1 and Observed_species were used to assess the number of species in the community, while the Shannon index was employed to evaluate species evenness within the community. The statistical results at the bottom of Table 5 reveal that both the group and time factors, their interaction, significantly impacted the Observed OTUs. Table 5 illustrates the effects of NCMCA treatment and composting time on the microbial community through three diversity indices (Observed OTUs, Shannon, and Chao1), the mean ± standard deviation of these indices, with lowercase letters within each index column indicating significant differences. Both group and time factors had a significant impact on the Shannon index, but there was no significant interaction between them. In contrast, group, time, and their interaction all exerted a significant influence on Chao1. Thus, composting time and NCMCA treatment, either individually or interactively, significantly affect microbial community diversity, and the treatment effect varies across different composting stages.

**TABLE 5:** Parametric tests with multiple-comparison corrections for α-diversity^*a*^

Group	Time	Observed OTUs	Shannon	Chao1
Control	D7	950.33 ± 5.51 b	7.39 ± 0.015 b	946.43 ± 14.00 b
	D28	986 ± 14 a	7.4 ± 0.072 b	990.37 ± 5.07 a
	D56	990.67 ± 19.43 a	7.85 ± 0.075 a	980.42 ± 29.80 ab
NCMCA	D7	986 ± 23.07 a	7.25 ± 0.096 b	982.74 ± 13.90 b
	D28	850 ± 22.72 b	7.32 ± 0.035 b	873.38 ± 11.30 c
	D56	1,026.67 ± 16.29 a	7.63 ± 0.068 a	1,050.76 ± 18.05 a
Group		*	***	ns
Time		***	***	***
Group*Time		***	ns	***

^*a*^ * means *P *< 0.05, *** means *P *< 0.001, and ns means non-significant (*P* > 0.05). Different letters indicate significant differences within the groups, while the same letters indicate no significant differences within the groups.

**Fig 4:**
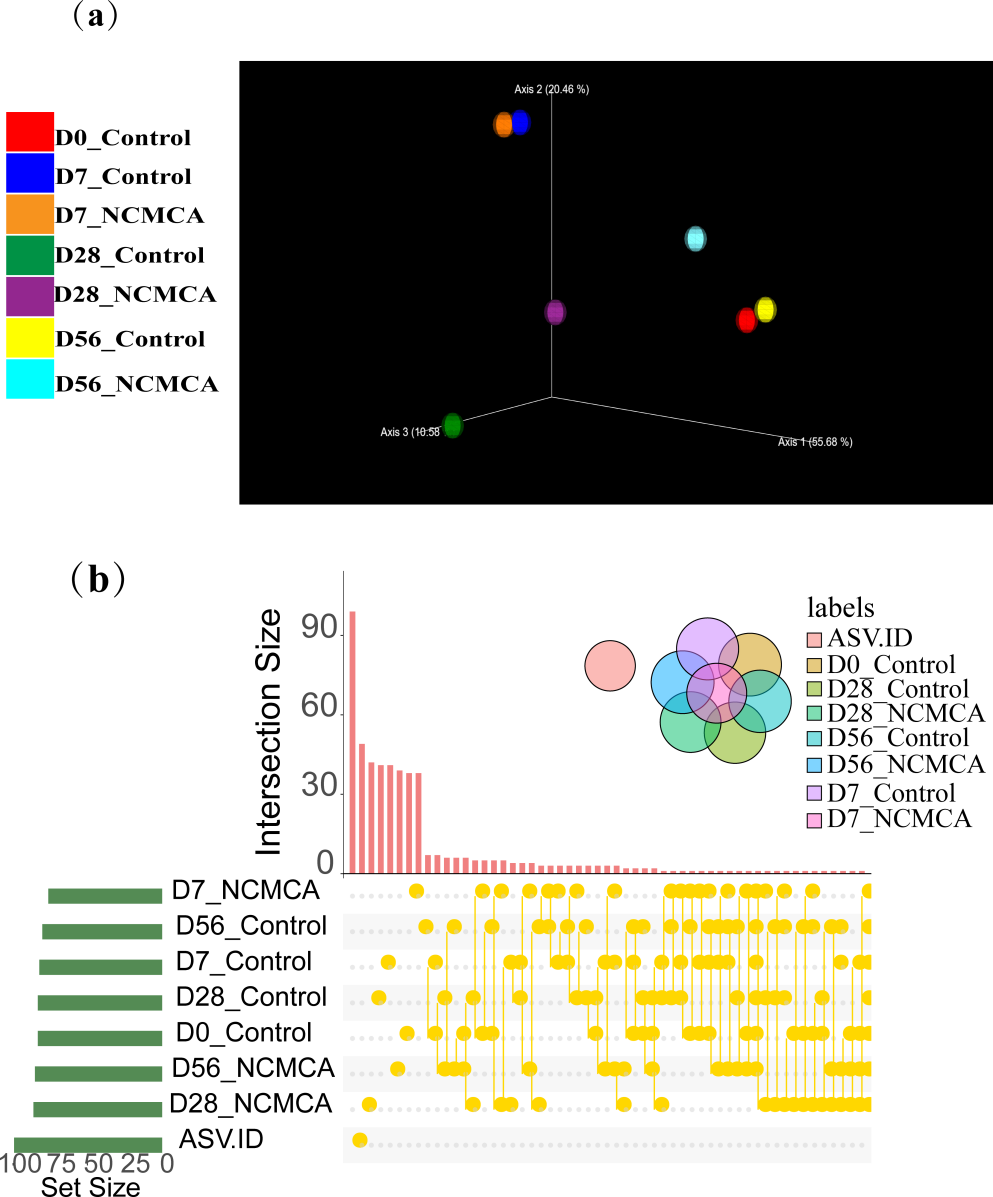
3D PCoA visualization (**a**) and ASV-based Upset plot (**b**).

β-diversity indicates the differences in species between different environmental communities. When combined with α-diversity, they form the overall diversity, which reflects the biological heterogeneity of a given environmental community. It is mainly used to determine the degree of community change in compost samples. As observed from Fig. 4a, the distance between the control at the onset (day 0) and the thermophilic stage (day 7) was relatively short, while the distance between the NCMCA treatment in the thermophilic stage was relatively long. This indicates that the community structure of the NCMCA treatment and the control was significantly different, suggesting that the addition of NCMCA can alter the biological structure of the community. On day 28, the control and the NCMCA treatment were closer to each other. At the end of composting, the community structure of the control and the NCMCA treatment was significantly different (*P* < 0.05), further demonstrating the effect of NCMCA on changing the community biological structure. Figure 4b presents an upset plot based on ASV data, illustrating the relationship between the numbers of shared and unique ASVs among microbial communities in different composting groups. There was a relatively small shared ASVs between control and NCMCA treatment on days 7, 28, and 56, respectively, indicating a minimal intersection. This suggests that NCMCA has a significant and persistent impact on the community structure. Within the same treatment, there were a few intersecting ASVs at different time points, suggesting the succession process of the community. The NCMCA treatment can enhance community richness, which is evident during the cooling (D28) and maturity (D56) stages of composting. These results provide community-level evidence for understanding how NCMCA treatment regulates the microbial community to achieve functions such as nitrogen conservation.

### Dynamic changes in microbial communities during composting

**Fig 5:**
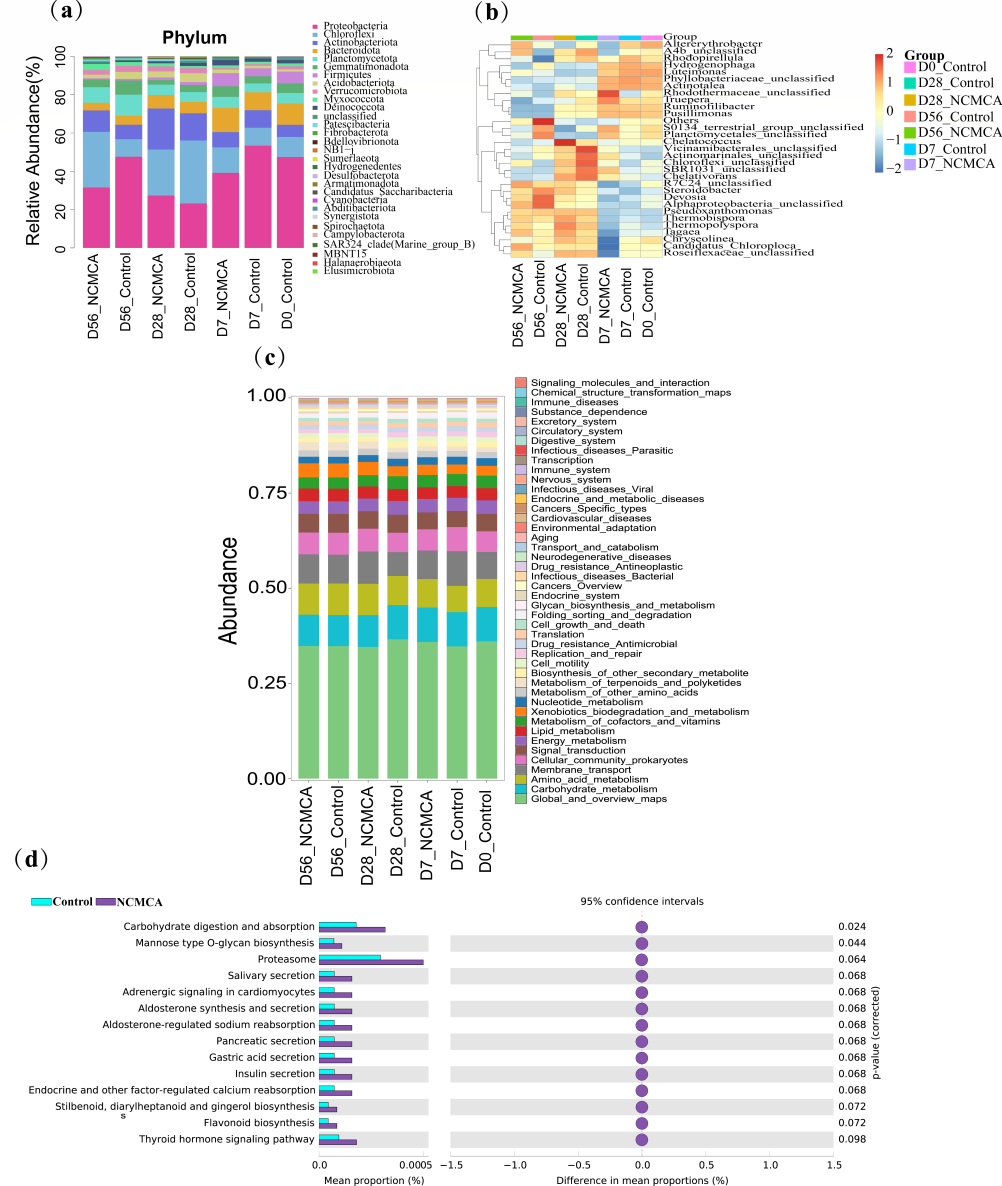
Relative abundance of bacteria at the phylum level (**a**) and genus level (**b**) and the outcomes of the gene function prediction at the second (**c**) and third levels (**d**). Note: Low-abundance, non-specific annotations are a common technical artifact of metagenomic prediction tools and do not reflect actual metabolic activity in the composting.

As depicted in Fig. 5a and b, an in-depth analysis was conducted on the relative abundances of the top 30 bacteria at the phylum and genus levels throughout the aerobic composting. The Proteobacteria, Chloroflexi, Actinobacteriota, Bacteroidota, Planctomycetota, and Gemmatimonadota emerged as the dominant phyla in agreement with previous studies (2, 43), which validates the consistency of microbial community patterns in similar composting environments. Figure 5a revealed that Proteobacteria held the highest proportion during the heating and maturation stages. On day 0, it accounted for 47.47% of the microbial community. During the thermophilic stage (day 7), its abundance reached 53.4% in the control, while in the NCMCA-inoculated group, it was 39.19%. By day 56, Proteobacteria accounted for 47.61% in the control, but only 31.59% in the NCMCA treatment. This significant decrease in the NCMCA treatment samples suggests that the inoculation of NCMCA had an adverse impact on the survival of Proteobacteria. This could be because during the thermophilic stage, thermostable bacteria such as Chloroflexi and Firmicutes outcompeted and replaced the mesophilic bacteria within the Proteobacteria phylum. This shift in dominance reflects the dynamic nature of microbial communities in response to environmental changes induced by the composting and the presence of NCMCA. After Proteobacteria, Bacteroidetes, and Chloroflexi were the next most prominent phyla, with relative abundances of 11.03% and 10.46%, respectively. Interestingly, the trend in the abundance of Chloroflexi was opposite to that of Proteobacteria. Compared to the control, the relative abundance of Chloroflexi in the NCMCA-treated samples gradually increased, reaching its peak on day 28. This increase is due to the unique metabolic capabilities of Chloroflexi, which possess diverse autotrophic metabolic pathways and efficiently degrade refractory OM (44). This allows Chloroflexi to thrive in the composting environment and contribute to the overall decomposition process. In the decomposition stage, Chloroflexi content in the NCMCA treatment significantly exceeded that in the control (*P* < 0.05). This disparity might be due to a synergistic effect between Chloroflexi and NCMCA, as reported in a previous study (2). This synergy likely enhances the ability of Chloroflexi to degrade complex organic compounds, further promoting the composting. Members of the Bacteroidetes phylum are known for their ability to degrade small-molecule substances by secreting a variety of enzymes that break down complex molecules of OM into simpler forms (9). Their relative abundance increased during the thermophilic and cooling phases of composting. This adaptability can be related to the diverse nature of Bacteroidetes members, which enables them to thrive across a wide range of temperatures (9). In contrast to the control, the abundance of Actinomycetes exhibited a decline in the initial phase of NCMCA-aided composting but increased in the middle and later stages due to denitrification (45). The presence of Actinomycetes is an indicator of favorable composting (46). Planctomycetota demonstrated a significant change in response to the variation of composting stages (44). The abundance of Gemmatimonadota initially increased and subsequently decreased. This group of microorganisms can produce hormone-like substances and secrete antibiotics to eliminate pathogenic microorganisms, thereby enhancing the quality of compost products (36).

At the genus level (Fig. 5b), in the thermophilic stage, the predominant genera in the control were *Luteimonas* (17.00%), *Hydrogenophaga* (5.99%), *Pusillimonas* (4.60%), *Ruminofilibacter* (3.24%), and *Candidatus_Chloroploca* (2.68%). In the NCMCA treatment, the dominant genera were *Luteimonas* (12.28%), *Ruminofilibacter* (6.63%), *Pusillimonas* (4.34%), *Hydrogenophaga* (3.50%), *Truepera* (2.75%), and *Actinotalea* (1.84%). *Luteimonas*, *Hydrogenophaga*, and *Pusillimonas* belong to Proteobacteria and play crucial regulatory roles in the regulation of nitrogen transformation genes (47). Their presence and abundance can significantly influence the nitrogen cycling processes during composting, affecting the availability of nitrogen for plants and the overall quality of the compost. On day 28, the dominant genera in the control were *Candidatus_Chloroploca* (5.74%), *Thermopolyspora* (5.00%), and *Pseudoxanthomonas* (3.91%). In the NCMCA treatment, they were *Candidatus_Chloroploca* (5.62%), *Thermopolyspora* (9.71%), *Pseudoxanthomonas* (4.32%), and *Thermobispora* (3.46%). The differences in the relative abundances of these genera between the two groups indicate that NCMCA inoculation can alter the composition of the microbial community at the genus level. In the maturation stage, the dominant bacteria in the control were *Devosia* (9.2%), *Pseudoxanthomonas* (3.48%), *Candidatus_Chloroploca* (3.03%), *Luteimonas* (2.92%), and *Steroidobacter* (2.36%). In the NCMCA treatment, the dominant genera included *Candidatus_Chloroploca* (18.82%), *Luteimonas* (4.74%), *Thermopolyspora* (3.99%), and *Pseudoxanthomonas* (2.66%). Overall, the inoculation of NCMCA was associated with an increase in the relative abundance of *Candidatus_Chloroploca* and *Ruminofilibacter*, while corresponding to a decrease in the relative abundance of *Devosia*, *Pusillimonas*, *Hydrogenophaga*, and other genera. These shifts in the relative abundance of dominant genera may have significant impacts on composting processes, such as influencing the degradation of OM and nitrogen cycling.

To further elucidate the alterations in the metabolic pathways of microbial communities in cow manure composting following the secondary inoculation of NCMCA, this study employed the PICRUSt2 functional prediction technology; the function prediction was conducted using PICRUSt2 v2.2.0. By default, the software calculates the nearest sequence taxon index (NSTI) for each amplicon sequence variant (ASV) and filters out ASVs with an NSTI value exceeding 2; the differential analysis and graph generation were carried out using STAMP. By leveraging the *16S rDNA* sequences of the sequenced bacterial genomes, the gene functional profiles of their common ancestors were inferred. Through a comparison with the KEGG database, the gene functions of the entire microbial community were predicted and analyzed. The outcomes of the gene function prediction at the second and third levels are presented. Figure 5c revealed the differences in metabolic pathways or functional characteristics between the treatment group and the control group from a functional perspective, explaining “how the community functions changed”; the functional metabolisms of both the control and the NCMCA treatment resembled those observed at day 0. The principal metabolic functions and their relative abundances were as follows: Global_and_overview_maps (ranging from 34.61% to 36.61%), Carbohydrate_metabolism (8.19%–9.02%), Amino_acid_metabolism (6.90%–8.24%), Membrane_transport (6.26%–9.10%), Cellular_community_prokaryotes (5.10%–6.34%), and Signal_transduction (4.21%–4.92%). Due to sequence homology or low-abundance matches across diverse microbial genomes, occasional non-specific annotations may emerge, such as “Adrenergic signaling in cardiomyocytes” that appear unrelated to composting, particularly for pathways with broad sequence associations. In our quantitative analysis, these non-specific pathways account for only a very low relative abundance (<0.5%) in the overall functional profile, far below the dominant pathways directly involved in composting processes. Further examinations at the third level (Fig. 5d) showed that the metabolic pathways related to carbohydrate digestion and absorption and mannose-type O-glycan biosynthesis in the NCMCA treatment were significantly more active than those in the control. Therefore, the NCMCA treatment altered the functional pathways during the critical stages of composting by modifying the microbial community structure, ultimately influencing composting efficiency and product quality.

**Fig 6:**
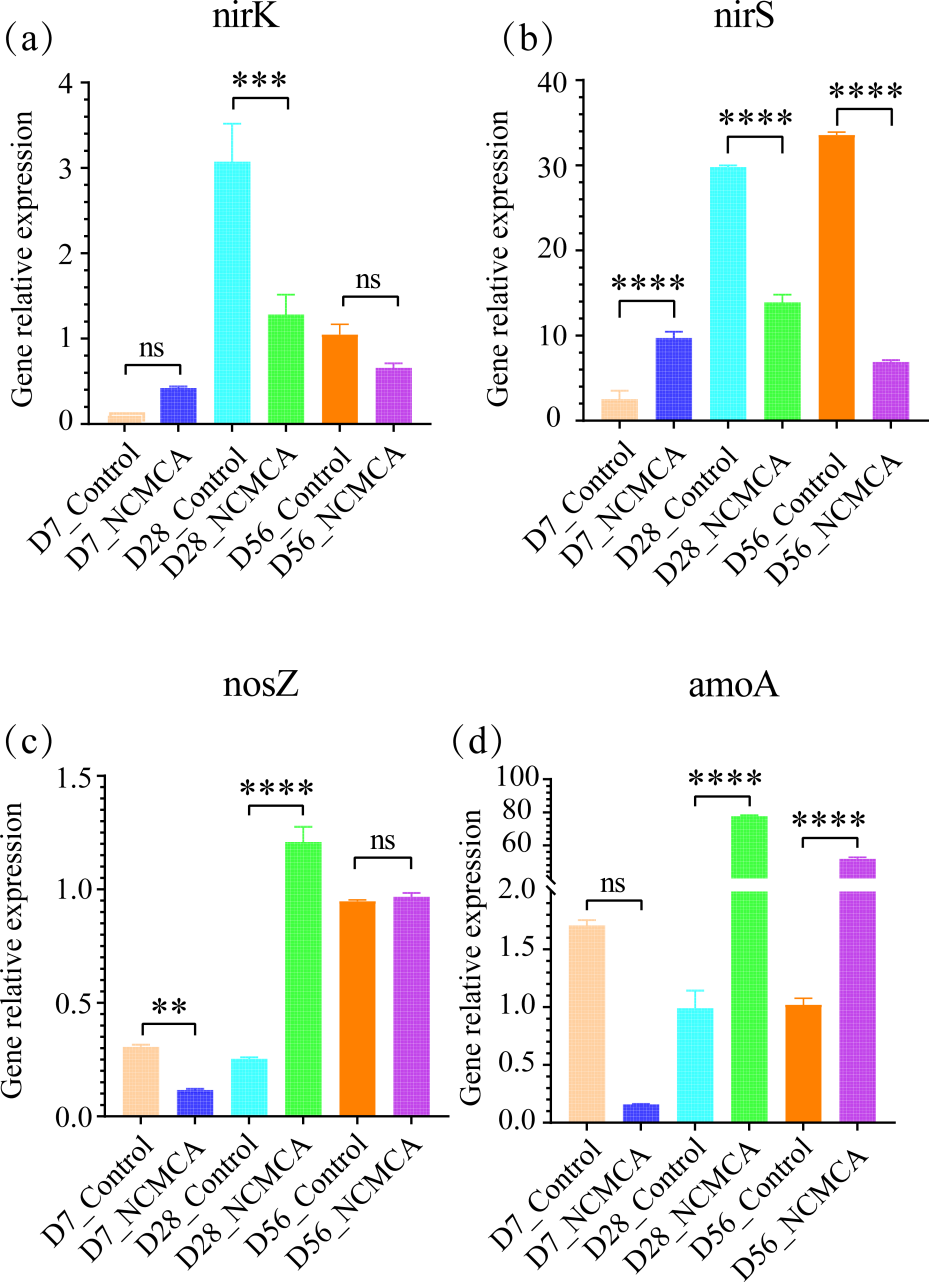
Gene relative abundance normalized to day 0: nirK (**a**), nirS (**b**), nosZ (**c**), and amoA (**d**). ** means *P* < 0.01, *** means *P* < 0.001, **** means *P* < 0.0001, and ns means non-significant.

To further verify the nitrification and denitrification, the abundance of the *nirS*, *nirK*, *nosZ*, and *amoA* genes was detected by qPCR using samples from day 0 as the baseline. As shown in Fig. 6a, no significant difference in the abundance of *nirK*-type denitrifying bacteria was observed during the thermophilic and maturation periods. However, during the cooling period, the NCMCA treatment showed significantly lower levels compared to the control treatment (*P* < 0.001), suggesting a potential for reducing N_2_O emissions (48). Compared with the control, the *nirS* gene in the NCMCA treatment (Fig. 6b) exhibited a significant increase during the thermophilic period (*P* < 0.0001), followed by a significant decrease during the cooling and maturation periods (*P* < 0.0001), which aligns with the findings of Xie J et al. (49); N_2_O reductase (*nosZ*) plays a key role in converting N_2_O into Nitrogen (N_2_), thereby reducing greenhouse gas emissions. As shown in Fig. 6c, the expression of *nosZ* significantly increased during the cooling period (*P* < 0.0001), further supporting its role in mitigating N_2_O emissions. Ammonia-oxidizing bacteria (*amoA*), as shown in Fig. 6d, were significantly more abundant in the NCMCA treatment during the cooling and maturation stages of composting (*P* < 0.0001). This suggests an enhanced capacity to convert NH_4_^+^ to NO_3_^−^ (50). These findings indicate that NCMCA contributes to nitrogen retention by inhibiting denitrification and promoting nitrification.

### Correlation and network analysis of dominant microbial communities

**Fig 7:**
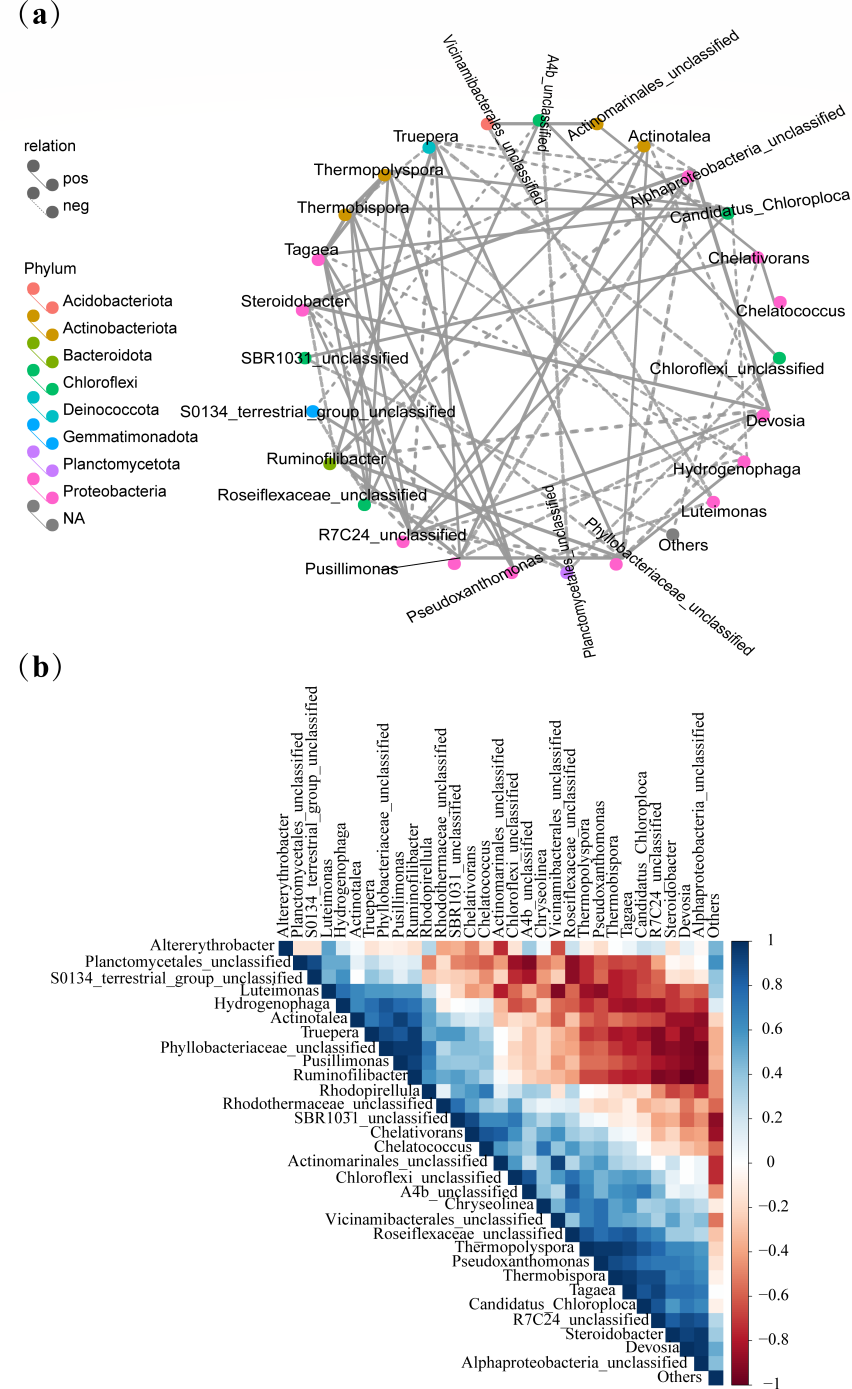
Correlation network analysis of bacteria at the phylum level (**a**) and genus level (**b**) during composting.

Symbiotic networks serve as powerful tools for depicting the intricate interactions among microorganisms. The role of each microorganism in the network can be inferred from its node attributes (51). In this study, Spearman’s rank correlation analysis was utilized to evaluate the abundances of the top 30 microorganisms at a specific taxonomic level. This analysis generated both *P* value matrices and correlation heat maps, providing a comprehensive view of the relationships among different microbial groups. In the network diagram, the color of each node represents the phylum level of the corresponding species (Fig. 7a). A connection between two nodes implies a significant correlation at the genus level. The size of a node is determined by two factors: the number of potential bacteria associated with it and the significance of the correlation, represented by the *P* value. Only correlations with a coefficient greater than 0.4 were considered statistically significant for interpreting the community structure, as per the criteria set in the literature (34). As shown in Fig. 7a, different genera of Proteobacteria exhibited strong positive correlations with various genera of Actinomycetes and Bacteroidetes. Conversely, they showed negative correlations with Chloroflexi, which is the dominant phylum during the cooling stage. The abundance of *Steroidobacter*, *Tagaea*, *Actinotalea*, *Devosia*, *Hydrogenophaga*, *Pusillimonas*, and *Luteimonas* was significantly correlated with that of other bacteria in the network. Figure 7b further corroborates these findings in that during the warming stage, *Luteimonas* demonstrated significant negative correlations with *Candidatus_Chloroploca*, *Thermopolyspora*, *Pseudoxanthomonas*, and *Devosia* in the maturation stage, with correlation coefficients of −0.64, −0.86, −0.89, and −0.57, respectively. *Luteimonas* is known to facilitate the decomposition of polysaccharides and OM (52). *Ruminofilibacter*, which was isolated from the rumen of ruminants, participates in the decomposition of hemicellulose (53). These two genera may have a synergistic effect on cellulose decomposition. *Thermopolyspora* and *Pseudoxanthomonas* showed positive correlations with *Devosia*, with correlation coefficients of 0.89 and 0.71, respectively. They also correlated positively with the dominant bacteria of *Hydrogenophaga*, *Ruminofilibacter*, *Pusillimonas*, and *Truepera*, with coefficients of 0.75, 0.56, 0.56, and 0.57, respectively. *Devosia* possesses bioremediation potential (54), while *Thermopolyspora* and *Pseudoxanthomonas* can co-degrade lignocellulose (55). In this context, *Devosia* may play a synergistic role in maintaining environmental stability during composting. Microbial correlation and network are vital to composting by ensuring efficient composting and high-quality output of compost products. This is achieved by influencing environmental physicochemical parameters, decomposition of OM, maturation process of compost, suppressing the proliferation of detrimental microorganisms, and enhancing the quality of compost products.

### Analysis of environmental factors

**Fig 8:**
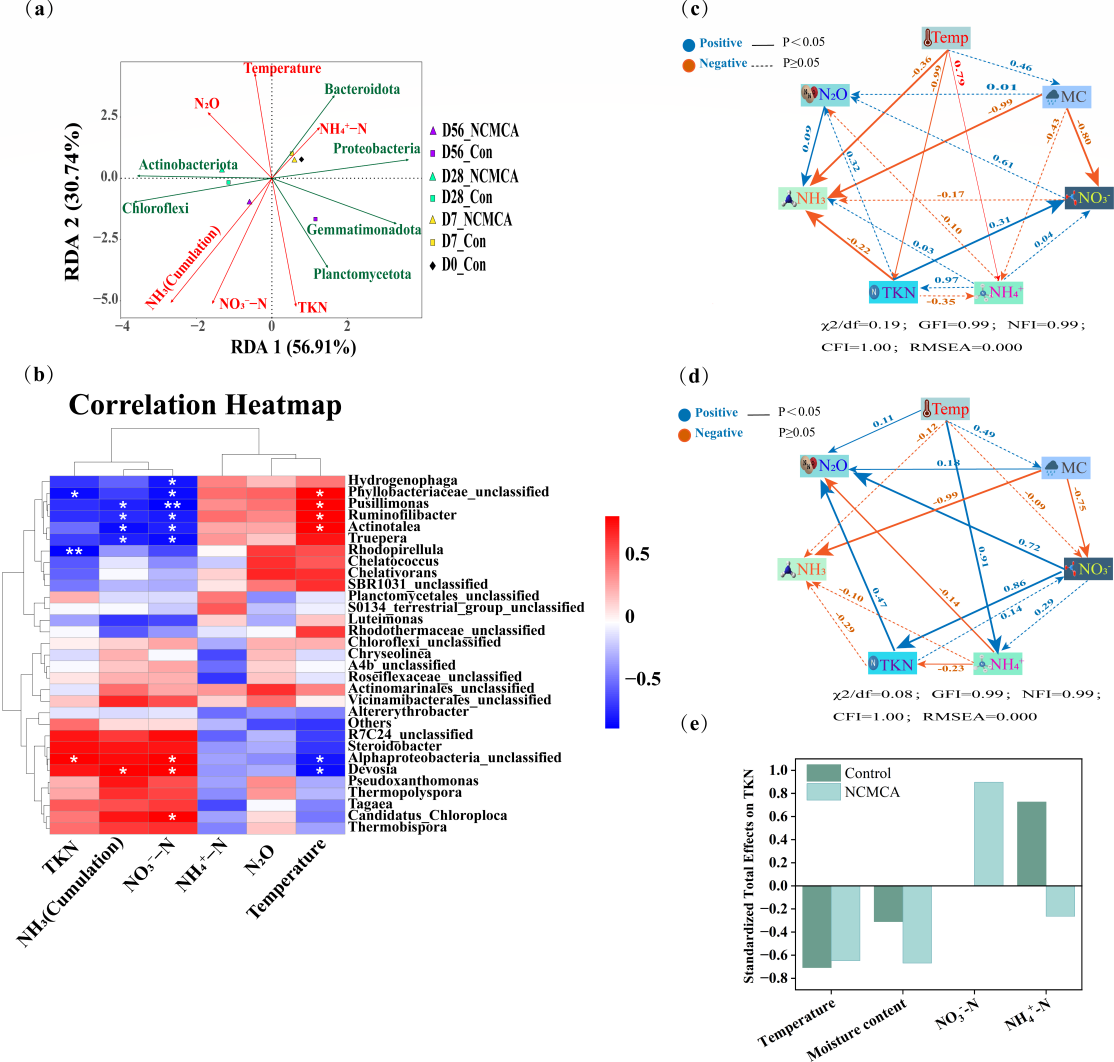
Correlation analysis of environmental factors and bacterial communities. (**a**) Redundancy Analysis (RDA) of the top six bacterial communities at the phylum level and environmental factors; each point represents a sample, and the closer the distance between two points, the higher the similarity of the community structure; the arrows represent the influencing factors, and the acute angle between the two factors indicates a positive correlation, while the obtuse angle indicates a negative correlation. The longer the ray, the greater the effect of the factor; the position of the projection point of the sample on the arrow represents the magnitude of the factor in the corresponding sample. (**b**) Correlation analysis of the top 30 bacterial communities at the genus level and environmental factors; rows represent genera, and columns represent environmental indicators. * means *P <* 0.05 and ** means *P* < 0.01. Red indicates positive correlation, and blue indicates negative correlation. The darker the color, the stronger the correlation. (**c**) SEM (Con). (**d**) SEM (NCMCA). The numbers on the straight line represent the size of the path coefficient. (**e**) Standard total effect of TKN.

Redundancy analysis (RDA) was utilized to comprehensively assess the impact of environmental factors on compost samples. From Fig. 8a, the selected environmental factors accounted for 87.65% of the variation, with RDA1 contributing 56.91% and RDA2 accounting for 30.74%. This indicates that the microbial community structure is intricately and closely related to the physicochemical characteristics of the feces (56). In this study, we attempted to unravel the relationships among the top six most abundant phyla (Fig. 8a), the top 30 genera (Fig. 8b), and environmental factors. In the two-dimensional sequence diagram depicting compost sample points and process factors, the positions of the initial composting stage (day 0) and the thermophilic stage (day 7) were not remarkably different. However, the positions of the same treatment at different times varied significantly, suggesting that the bacterial community structure was substantially affected by the composting in the early stages. From day 0 to the maturity stage (day 56), the positions of different treatments at the same time were relatively close. Nevertheless, at the maturity stage, the positions diverged considerably, indicating that the community structure at this stage was distinctively different, as shown in Fig. 4a, further validating the dynamic nature of the microbial community during composting. The RDA results revealed that during the thermophilic stage (day 7), Bacteroidota, Chloroflexi, Proteobacteria, and Actinobacteriota were positively correlated with temperature and N_2_O emissions. This indicated that elevated temperatures were conducive to the increase in the abundance of Bacteroidota and Actinobacteriota, which in turn impacted the cumulative NH_3_ emissions and N_2_O emissions. In the later phases of composting, Planctomycetota, Chloroflexi, and Gemmatimonadota had a positive relationship with TKN, cumulative NH_3_ emissions, and NO_3_^-^-N. These correlations demonstrated that these microbial groups are important in nitrogen transformation and the release of nitrogen-containing gases during different stages of composting.

To explore in depth the influence of environmental factors on the bacterial community composition, Pearson correlation analysis was conducted between the top 30 dominant genera and environmental factors (Fig. 8b). The results indicated that *Pusillimonas* (*P* = 0.016), *Ruminofilibacter* (*P* = 0.016), and *Actinotalea* (*P* = 0.048) were significantly positively correlated with temperature. In contrast, *Devosia* (*P* = 0.024) showed a negative correlation with temperature. Additionally, *Pusillimonas* (*P* = 0.008, 0.033), *Ruminofilibacter* (*P* = 0.027, 0.049), *Actinotalea* (*P* = 0.048, 0.024), and *Truepera* (*P* = 0.024, 0.048) were inversely related to the cumulative emissions of NO_3_^-^-N and NH_3_, which was beneficial for nitrogen preservation. On the contrary, *Devosia* (*P* = 0.048, 0.048) was positively correlated with their contents. Moreover, the content of NO_3_^-^-N was negatively correlated with *Hydrogenophaga* (*P* = 0.034) and positively correlated with *Candidatus_Chloroploca* (*P* = 0.048). *Rhodopirellula* (*P* = 0.007) was significantly negatively correlated with TKN content. Among them, *Pusillimonas*, *Ruminofilibacter*, *Devosia*, and *Hydrogenophaga* all belong to the Proteobacteria phylum. Many bacteria in the Proteobacteria and Bacteroidota phyla play crucial functions in the transfer and transformation of nitrogen during aerobic composting (9). The nitrogen sources required for plant absorption are all derived from the decomposition of OM by these bacteria (8). This might be the reason for the dominance of Proteobacteria phylum during the thermophilic stage. Actinotalea belongs to the Actinobacteria phylum, and its correlation with environmental factors also reflects the role it plays in the complex ecosystem of composting.

Structural equation modeling (SEM) was deployed to uncover the underlying mechanism between each factor and nitrogen transformation after NCMCA inoculation (Fig. 8c and d). Compared with the control, the inoculation of NCMCA strengthened the correlation between TKN and N_2_O, as well as between TKN and NH_4_^+^. At the same time, it reduced the correlation between TKN and NH_3_, as well as between temperature and TKN. Nitrate and TKN were significantly correlated (*P* < 0.001, λ = 0.86)*,* indicating that the inoculation of NCMCA could effectively promote the transformation of nitrate to TKN. From the standard comprehensive effect (Fig. 8e), the inoculation of NCMCA could significantly reduce the MC, which might cause the infiltration of nitrate (9). Additionally, the significant increase in nitrate content and the decrease in ammonium content suggested that the inoculation of NCMCA promoted the nitrification. The reduction of ammonium ions decreased the volatilization of ammonia (57), and the increase in NO_3_^-^ content could enhance the TKN content (31), thus achieving the purpose of conservation of nitrogen. The model fit indices (Table 6) indicate that the structure of this model fits excellently. Moreover, this model is constructed based on existing literature or theoretical frameworks, with clear clarification of the causal relationships between variables (9, 57).

**TABLE 6:** Model fit indices of the structural equation model

Common indicators	Absolute fit indices			
	χ, χ²	χ/df, χ²/df	Root mean square error of approximation, RMSEA	Standardized root mean square residual, SRMR
Value (Control)	0.381	0.190	0.000	0.0059
Value (NCMCA)	0.321	0.080	0.000	0.0047

Common indicators	Relative fit indices			
	Comparative fit index, CFI		Incremental fit index, IFI	
Value (Control)	1.000		1.000	
Value (NCMCA)	1.000		1.000	

Common indicators	Parsimonious fit indices			
	Akaike information criterion, AIC		Bayesian information criterion, BIC	
Value (Control)	52.381		60.248	
Value (NCMCA)	48.321		55.583	

In conclusion, the secondary inoculation of NCMCA could rapidly initiate the composting process by prolonging the thermophilic period, increasing the peak temperature, modulating the pH and EC of the pile, and reducing the MC. These changes in physicochemical factors induced alterations within the structure of the bacterial community. When considering the phylum level, it decreased the abundance of Proteobacteria and increased the content of Chloroflexi, Actinobacteria, and Bacteroidetes. At the genus level, it favored the reproduction of *Candidatus_Chloroploca* and *Ruminofilibacter*, while decreasing the reproduction of *Devosia*, *Pusillimonas*, and *Hydrogenophaga*. As illustrated in Fig. 8e, after the secondary inoculation of NCMCA, the emission of ammonia was reduced, with this reduction associated with a negative correlation between NCMCA and the relative abundance of *Devosia*, as well as a positive correlation with *Ruminofilibacter*, suggesting potential synergistic interactions. The content of NO_3_^-^-N was increased, which coincided with the promoted relative abundance of *Candidatus_Chloroploca* and reduced abundance of *Pusillimonas* and *Hydrogenophaga.* These correlations may contribute to the observed increase in TKN content. Additionally, the decrease in MC likely reduced the infiltration of nitrate, thereby increasing the nitrate content. This implied that the secondary inoculation of NCMCA enabled the composting to start up rapidly under low-temperature conditions, effectively altered the bacterial community structure, significantly reduced ammonia emissions, increased the TKN content, and effectively retained nitrogen in the composting.

### Conclusion

**Fig 9:**
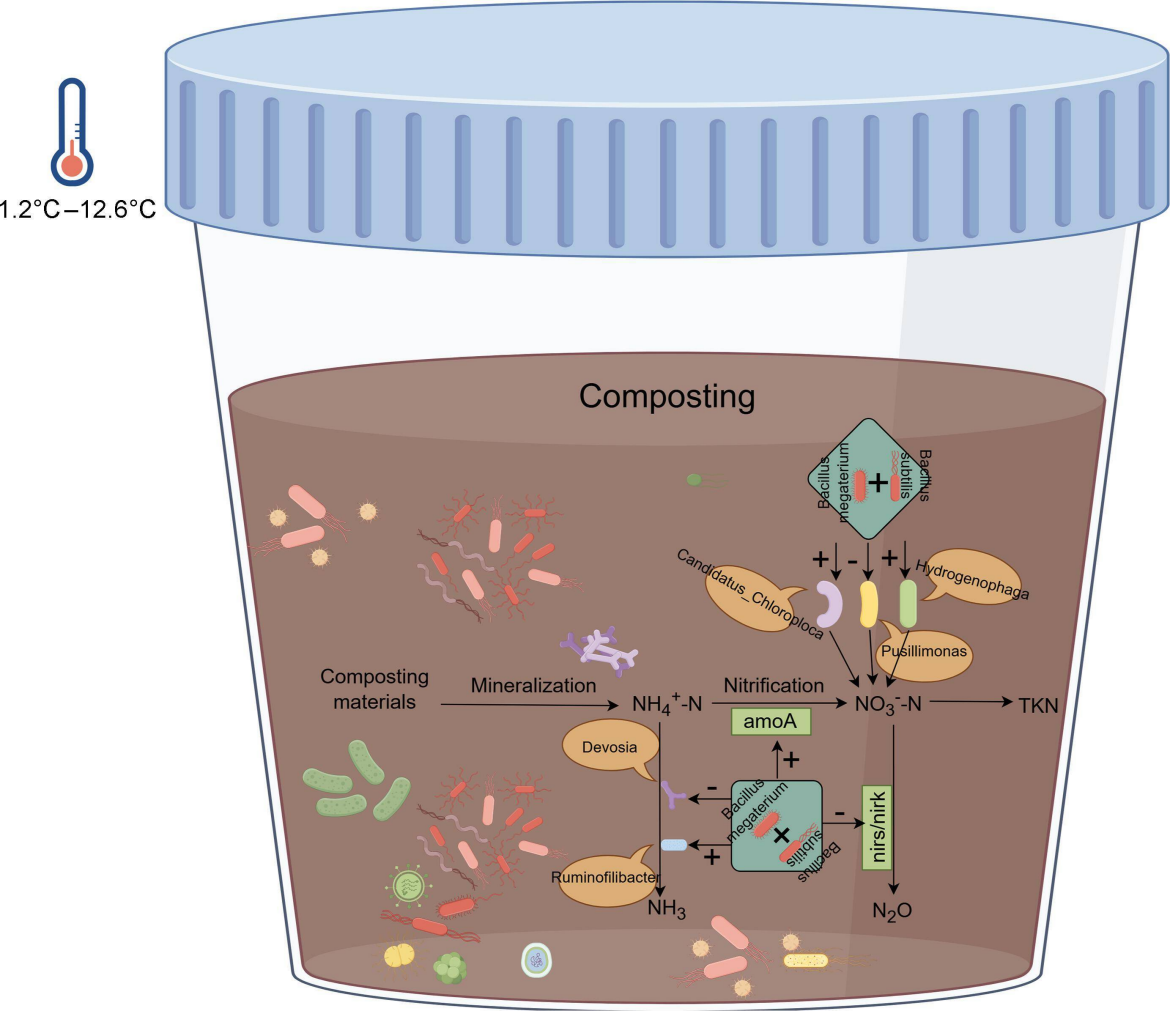
Mechanism diagram.

The present study, conducted under low temperature conditions, showed that inoculating 5% NCMCA on day 1 and day 28 increased the peak temperature, prolonged the thermophilic period, and retained nitrogen of composting, compared with the control. As shown in Fig. 9, NCMCA promoted nitrogen retention, enhanced nitrification, and inhibited denitrification by stimulating ammonia-oxidizing bacteria (amoA) and suppressing nirs/nirk-type denitrifying bacteria, thereby reducing NH₃ emissions; this effect was negatively correlated with the relative abundance of Devosia and positively correlated with Ruminofilibacter, suggesting potential interactive effects. The observed increase in TKN content in the NCMCA treatment was accompanied by a higher relative abundance of *Candidatus_Chloroploca* and lower abundances of *Pusillimonas* and *Hydrogenophaga.* These community shifts may be related to the significantly higher activity of metabolic pathways associated with carbohydrate digestion and absorption, as well as mannose-type O-glycan biosynthesis, compared to the control. Extensive interconnections among bacterial communities were noted, which provide a basis for the degradation of OM and promote the advancement of composting. These findings suggest promising application prospects for the novel composting strategy utilizing NCMCA in cold regions. This study primarily investigated conditions under which the ambient temperature ranged from 1.2 to 12.6°C, achieving preliminary success. In northern China, winter temperatures typically fall below 0°C across most areas. Therefore, whether NCMCA remains effective under sub-zero temperature conditions warrants further investigation. This question constitutes one of the key research priorities for our research treatment in future studies. Besides, this study did not cover a wider range of NCMCA dosage gradients, such as 1% and 10% (vol/wt), and thus could not directly verify the optimal application ratio, which represents a limitation of this study. Future research can further set up multi-dosage gradient experiments, combining composting efficiency with economic analysis to determine the optimal application concentration of NCMCA in different low-temperature environments, to provide more refined parameter support for practical applications.

## Data Availability

The *16S rDNA* amplicon sequencing data generated in this study have been deposited in the NCBI Sequence Read Archive (SRA) (https://www.ncbi.nlm.nih.gov/sra/). The BioProject accession number is PRJNA1321306, BioSample accession numbers are SAMN51193537–SAMN51193543, and SRA accession numbers are SRR35295313–SRR35295307.

## References

[1] Naz S, Fazio F, Habib SS, Nawaz G, Attaullah S, Ullah M, Hayat A, Ahmed I. 2022. Incidence of heavy metals in the application of fertilizers to crops (wheat and rice), a fish (common carp) pond and a human health risk assessment. Sustainability 14:13441. doi:10.3390/su142013441

[2] Xu Z, Li G, Huda N, Zhang B, Wang M, Luo W. 2020. Effects of moisture and carbon/nitrogen ratio on gaseous emissions and maturity during direct composting of cornstalks used for filtration of anaerobically digested manure centrate. Bioresour Technol 298:122503. doi:10.1016/j.biortech.2019.122503 31837581

[3] Wu X, Gao R, Tian X, Hou J, Wang Y, Wang Q, Tang DKH, Yao Y, Zhang X, Wang B, Yang G, Li H, Li R. 2024. Co-composting of dewatered sludge and wheat straw with newly isolated Xenophilus azovorans: carbon dynamics, humification, and driving pathways. J Environ Manage 365:121613. doi:10.1016/j.jenvman.2024.121613 38944964

[4] Wang Y, Yi G, Zhang W, Hou J, Daniel Tang KH, Wang B, Li H, Wang Q, Abdelrahman H, Zhang T, Shaheen SM, Li R. 2025. Ferrous salts accelerate humification and reduced carbon emissions during the co-composting of hoggery slurry and wheat husks: new insights into their biotic and abiotic functions and mechanisms. Chem Eng J 512:162570. doi:10.1016/j.cej.2025.162570

[5] Wu X, Zhao X, Yi G, Zhang W, Gao R, Tang DKH, Xiao R, Zhang Z, Yao Y, Li R. 2024. Promoting nitrogen conversion in aerobic biotransformation of swine slurry with the co-application of manganese sulfate and biochar. J Environ Manage 356:120604. doi:10.1016/j.jenvman.2024.120604 38518501

[6] Hoang HG, Thuy BTP, Lin C, Vo D-V, Tran HT, Bahari MB, Le VG, Vu CT. 2022. The nitrogen cycle and mitigation strategies for nitrogen loss during organic waste composting: a review. Chemosphere 300:134514. doi:10.1016/j.chemosphere.2022.134514 35398076

[7] Jiao M, Yue F, Ren X, Zhan X, Xu W, Tang DKH, Xiao R, Li R. 2024. Enhanced humification attributed by the integration of Fenton reagent and oxalic acid during a co-composting of swine manure and corn straw: Impacts and the possible mechanisms. Chem Eng J 498:155579. doi:10.1016/j.cej.2024.155579

[8] Li C, Li H, Yao T, Su M, Li J, Liu Z, Xin Y, Wang L, Chen J, Gun S. 2020. Effects of microbial inoculation on enzyme activity, available nitrogen content, and bacterial succession during pig manure composting. Bioresour Technol 306:123167. doi:10.1016/j.biortech.2020.123167 32192957

[9] Tian X, Gao R, Li Y, Liu Y, Zhang X, Pan J, Tang KHD, Scriber II KE, Amoah ID, Zhang Z, Li R. 2023. Enhancing nitrogen conversion and microbial dynamics in swine manure composting process through inoculation with a microbial consortium. J Clean Prod 423:138819. doi:10.1016/j.jclepro.2023.138819

[10] Zhou Z, Shi X, Bhople P, Jiang J, Chater CCC, Yang S, Perez-Moreno J, Yu F, Liu D. 2024. Enhancing C and N turnover, functional bacteria abundance, and the efficiency of biowaste conversion using Streptomyces-Bacillus inoculation. J Environ Manage 358:120895. doi:10.1016/j.jenvman.2024.120895 38626487

[11] Jia X, Zhao K, Zhao J, Lin C, Zhang H, Chen L, Chen J, Fang Y. 2023. Degradation of poly(butylene adipate-co-terephthalate) films by Thermobifida fusca FXJ-1 isolated from compost. J Hazard Mater 441:129958. doi:10.1016/j.jhazmat.2022.129958 36122523

[12] Beaulieu R, López-Mondéjar R, Tittarelli F, Ros M, Pascual JA. 2011. qRT-PCR quantification of the biological control agent Trichoderma harzianum in peat and compost-based growing media. Bioresour Technol 102:2793–2798. doi:10.1016/j.biortech.2010.09.120 21030250

[13] Meng Y, Wei Y, Jin M, Zhang Y, Zhang S. 2024. Straw degradation enhanced in Thermomyces lanuginosus by transferring AgCMCase from Aspergillus glaucus. Bioresour Technol 413:131431. doi:10.1016/j.biortech.2024.131431 39241812

[14] Zhuo Cai J, Lan Yu Y, Biao Yang Z, Xun Xu X, Chun Lv G, Lian Xu C, Yin Wang G, Qi X, Li T, Bon Man Y, Hung Wong M, Cheng Z. 2024. Synergistic improvement of humus formation in compost residue by fenton-like and effective microorganism composite agents. Bioresour Technol 400:130703. doi:10.1016/j.biortech.2024.130703 38631654

[15] Huang S, Dong Q, Che S, Li R, Tang KHD. 2025. Bioplastics and biodegradable plastics: a review of recent advances, feasibility and cleaner production. Sci Total Environ 969:178911. doi:10.1016/j.scitotenv.2025.178911 40022973

[16] Miri S, Robert T, Davoodi SM, Brar SK, Martel R, Rouissi T, Lauzon J-M. 2023. Evaluation of scale-up effect on cold-active enzyme production and biodegradation tests using pilot-scale bioreactors and a 3D soil tank. J Hazard Mater 450:131078. doi:10.1016/j.jhazmat.2023.131078 36848843

[17] Abdellah YAY, Li T, Chen X, Cheng Y, Sun S, Wang Y, Jiang C, Zang H, Li C. 2021. Role of psychrotrophic fungal strains in accelerating and enhancing the maturity of pig manure composting under low-temperature conditions. Bioresour Technol 320:124402. doi:10.1016/j.biortech.2020.124402 33212385

[18] Barria C, Malecki M, Arraiano CM. 2013. Bacterial adaptation to cold. Microbiology (Reading, Engl) 159:2437–2443. doi:10.1099/mic.0.052209-0 24068238

[19] Fan Y, Yu K, Zheng H, Chen Y, Zhao R, Li Y, Zheng Z. 2023. A high-yielding strain of indole-3-acetic acid isolated from food waste compost: metabolic pathways, optimization of fermentation conditions, and application. Environ Technol 44:4199–4209. doi:10.1080/09593330.2022.2082889 35678156

[20] Su J, Zhou K, Chen W, Xu S, Feng Z, Chang Y, Ding X, Zheng Y, Tao X, Zhang A, Wang Y, Li J, Ding G, Wei Y. 2024. Enhanced organic degradation and microbial community cooperation by inoculating Bacillus licheniformis in low temperature composting. J Environ Sci (China) 143:189–200. doi:10.1016/j.jes.2023.08.037 38644016

[21] Xie F, Thiri M, Wang H. 2021. Simultaneous heterotrophic nitrification and aerobic denitrification by a novel isolated Pseudomonas mendocina X49. Bioresour Technol 319:124198. doi:10.1016/j.biortech.2020.124198 33038648

[22] De Maayer P, Anderson D, Cary C, Cowan DA. 2014. Some like it cold: understanding the survival strategies of psychrophiles. EMBO Rep 15:508–517. doi:10.1002/embr.201338170 24671034 PMC4210084

[23] Xie XY, Zhao Y, Sun QH, Wang XQ, Cui HY, Zhang X, Li YJ, Wei ZM. 2017. A novel method for contributing to composting start-up at low temperature by inoculating cold-adapted microbial consortium. Bioresour Technol 238:39–47. doi:10.1016/j.biortech.2017.04.036 28433916

[24] Zhou S, Geng B, Li M, Li Z, Liu X, Guo H. 2021. Comprehensive analysis of environmental factors mediated microbial community succession in nitrogen conversion and utilization of ex situ fermentation system. Sci Total Environ 769:145219. doi:10.1016/j.scitotenv.2021.145219 33486184

[25] Zhao B, Cao X, Cai Z, Zhang L, Li D, Zhang H, Li S, Sun X. 2023. Improving suppressive activity of compost on phytopathogenic microbes by inoculation of antagonistic microorganisms for secondary fermentation. Bioresour Technol 367:128288. doi:10.1016/j.biortech.2022.128288 36370939

[26] Liu X, Zubair M, Kong L, Shi Y, Zhou H, Tong L, Zhu R, Lv Y, Li Z. 2023. Shifts in bacterial diversity characteristics during the primary and secondary fermentation stages of bio-compost inoculated with effective microorganisms agent. Bioresour Technol 382:129163. doi:10.1016/j.biortech.2023.129163 37224888

[27] Zhu N, Zhu Y, Kan Z, Li B, Cao Y, Jin H. 2021. Effects of two-stage microbial inoculation on organic carbon turnover and fungal community succession during co-composting of cattle manure and rice straw. Bioresour Technol 341:125842. doi:10.1016/j.biortech.2021.125842 34469819

[28] Liu X, Kong L, Tong L, Zackariah GSK, Zhu R, Li Z, Lv Y. 2025. Inoculation with effective microorganisms agent enhanced fungal diversity in the secondary fermentation process. J Environ Manage 373:123985. doi:10.1016/j.jenvman.2024.123985 39752954

[29] Siu-Rodas Y, Calixto-Romo M de L, Guillén-Navarro K, Sánchez JE, Zamora-Briseño JA, Amaya-Delgado L. 2018. Bacillus subtilis with endocellulase and exocellulase activities isolated in the thermophilic phase from composting with coffee residues. Rev Argent Microbiol 50:234–243. doi:10.1016/j.ram.2017.08.005 29289440

[30] Zhao Y, Mao X, Zhang M, Yang W, Di HJ, Ma L, Liu W, Li B. 2021. The application of Bacillus Megaterium alters soil microbial community composition, bioavailability of soil phosphorus and potassium, and cucumber growth in the plastic shed system of North China. Agric Ecosyst Environ 307:107236. doi:10.1016/j.agee.2020.107236

[31] Xu Z, Li R, KuoK Ho Tang D, Zhang X, Zhang X, Liu H, Quan F. 2024. Enhancing nitrogen transformation and humification in cow manure composting through psychrophilic and thermophilic nitrifying bacterial consortium inoculation. Bioresour Technol 413:131507. doi:10.1016/j.biortech.2024.131507 39303947

[32] Xu Z, Li R, Zhang X, Liu J, Xu X, Wang S, Lan T, Zhang K, Gao F, He Q, Pan J, Quan F, Zhang Z. 2023. Mechanisms and effects of novel ammonifying microorganisms on nitrogen ammonification in cow manure waste composting. Waste Manag 169:167–178. doi:10.1016/j.wasman.2023.07.009 37442037

[33] Kalamdhad AS, Singh YK, Ali M, Khwairakpam M, Kazmi AA. 2009. Rotary drum composting of vegetable waste and tree leaves. Bioresour Technol 100:6442–6450. doi:10.1016/j.biortech.2009.07.030 19679465

[34] Xu Z, Li R, Liu T, Zhang G, Wu S, Xu K, Zhang Y, Wang Q, Kang J, Zhang Z, Quan F, Zhang Y. 2022. Effect of inoculation with newly isolated thermotolerant ammonia-oxidizing bacteria on nitrogen conversion and microbial community during cattle manure composting. J Environ Manage 317:115474. doi:10.1016/j.jenvman.2022.115474 35751273

[35] Jiang J, Liu X, Huang Y, Huang H. 2015. Inoculation with nitrogen turnover bacterial agent appropriately increasing nitrogen and promoting maturity in pig manure composting. Waste Manag 39:78–85. doi:10.1016/j.wasman.2015.02.025 25769536

[36] Li R, Xu K, Ali A, Deng H, Cai H, Wang Q, Pan J, Chang CC, Liu H, Zhang Z. 2020. Sulfur-aided composting facilitates ammonia release mitigation, endocrine disrupting chemicals degradation and biosolids stabilization. Bioresour Technol 312:123653. doi:10.1016/j.biortech.2020.123653 32531732

[37] Singh J, Kalamdhad AS. 2013. Assessment of bioavailability and leachability of heavy metals during rotary drum composting of green waste (Water hyacinth). Ecol Eng 52:59–69. doi:10.1016/j.ecoleng.2012.12.090

[38] Sun Q, Wu D, Zhang Z, Zhao Y, Xie X, Wu J, Lu Q, Wei Z. 2017. Effect of cold-adapted microbial agent inoculation on enzyme activities during composting start-up at low temperature. Bioresour Technol 244:635–640. doi:10.1016/j.biortech.2017.08.010 28810218

[39] Cui P, Liao H, Bai Y, Li X, Zhao Q, Chen Z, Yu Z, Yi Z, Zhou S. 2019. Hyperthermophilic composting reduces nitrogen loss via inhibiting ammonifiers and enhancing nitrogenous humic substance formation. Sci Total Environ 692:98–106. doi:10.1016/j.scitotenv.2019.07.239 31340193

[40] Nordahl SL, Preble CV, Kirchstetter TW, Scown CD. 2023. Greenhouse gas and air pollutant emissions from composting. Environ Sci Technol 57:2235–2247. doi:10.1021/acs.est.2c05846 36719708 PMC9933540

[41] Wang S, Zeng Y. 2018. Ammonia emission mitigation in food waste composting: a review. Bioresour Technol 248:13–19. doi:10.1016/j.biortech.2017.07.050 28736141

[42] Wang Z, Ding Y, Ren X, Xie J, Kumar S, Zhang Z, Wang Q. 2022. Effect of micronutrient selenium on greenhouse gas emissions and related functional genes during goat manure composting. Bioresour Technol 349:126805. doi:10.1016/j.biortech.2022.126805 35131460

[43] Wei H, Wang L, Hassan M, Xie B. 2018. Succession of the functional microbial communities and the metabolic functions in maize straw composting process. Bioresour Technol 256:333–341. doi:10.1016/j.biortech.2018.02.050 29459320

[44] de Gannes V, Eudoxie G, Hickey WJ. 2013. Prokaryotic successions and diversity in composts as revealed by 454-pyrosequencing. Bioresour Technol 133:573–580. doi:10.1016/j.biortech.2013.01.138 23475177

[45] Huang Y, Yang H, Li K, Meng Q, Wang S, Wang Y, Zhu P, Niu Q, Yan H, Li X, Li Q. 2022. Red mud conserved compost nitrogen by enhancing nitrogen fixation and inhibiting denitrification revealed via metagenomic analysis. Bioresour Technol 346:126654. doi:10.1016/j.biortech.2021.126654 34979278

[46] Sundberg C, Yu D, Franke-Whittle I, Kauppi S, Smårs S, Insam H, Romantschuk M, Jönsson H. 2013. Effects of pH and microbial composition on odour in food waste composting. Waste Manag 33:204–211. doi:10.1016/j.wasman.2012.09.017 23122203 PMC3520005

[47] Liu N, Liu Z, Wang K, Zhao J, Fang J, Liu G, Yao H, Pan J. 2024. Comparison analysis of microbial agent and different compost material on microbial community and nitrogen transformation genes dynamic changes during pig manure compost. Bioresour Technol 395:130359. doi:10.1016/j.biortech.2024.130359 38272144

[48] Liu N, Liao P, Zhang J, Zhou Y, Luo L, Huang H, Zhang L. 2020. Characteristics of denitrification genes and relevant enzyme activities in heavy-metal polluted soils remediated by biochar and compost. Sci Total Environ 739:139987. doi:10.1016/j.scitotenv.2020.139987 32535466

[49] Xie J, Gu J, Wang X, Hu T, Sun W, Song Z, Zhang K, Lei L, Wang J, Sun Y. 2023. Response characteristics of denitrifying bacteria and denitrifying functional genes to woody peat during pig manure composting. Bioresour Technol 374:128801. doi:10.1016/j.biortech.2023.128801 36842510

[50] Guo H, Gu J, Wang X, Yu J, Nasir M, Zhang K, Sun W. 2020. Microbial driven reduction of N_2_O and NH_3_ emissions during composting: effects of bamboo charcoal and bamboo vinegar. J Hazard Mater 390:121292. doi:10.1016/j.jhazmat.2019.121292 31810805

[51] Meng L, Xu C, Wu F, Huhe. 2022. Microbial co-occurrence networks driven by low-abundance microbial taxa during composting dominate lignocellulose degradation. Sci Total Environ 845:157197. doi:10.1016/j.scitotenv.2022.157197 35839876

[52] Dong S, Wei Y, Yu Q, Gao Y, Chen H, Zhou K, Cheng M, Wang B, Wei Y, Hu X. 2024. Inoculating functional bacteria improved the humification process by regulating microbial networks and key genera in straw composting by adding different nitrogen sources. Bioresour Technol 393:130022. doi:10.1016/j.biortech.2023.130022 37979883

[53] Yang X, Li R, Wang J, Xu W, Wang Y, Yi G, Zhang X, Zhu J, Mazarji M, Syed A, Bahkali AH, Zhang Z, Pan J. 2023. Exploring carbon conversion and balance with magnetite-amended during pig manure composting. Bioresour Technol 388:129707. doi:10.1016/j.biortech.2023.129707 37659668

[54] Jiang F, Jiang Z, Huang J, Tang P, Cui J, Feng W, Yu C, Fu C, Lu Q. 2023. Exploration of potential driving mechanisms of Comamonas testosteroni in polycyclic aromatic hydrocarbons degradation and remodelled bacterial community during co-composting. J Hazard Mater 458:132032. doi:10.1016/j.jhazmat.2023.132032 37451101

[55] Mei J, Li B, Su L, Zhou X, Duan E. 2022. Effects of potassium persulfate on nitrogen loss and microbial community during cow manure and corn straw composting. Bioresour Technol 363:127919. doi:10.1016/j.biortech.2022.127919 36089132

[56] Wei Y, Wu D, Wei D, Zhao Y, Wu J, Xie X, Zhang R, Wei Z. 2019. Improved lignocellulose-degrading performance during straw composting from diverse sources with actinomycetes inoculation by regulating the key enzyme activities. Bioresour Technol 271:66–74. doi:10.1016/j.biortech.2018.09.081 30265954

[57] Meng L, Li W, Zhang S, Wu C, Wang K. 2016. Effects of sucrose amendment on ammonia assimilation during sewage sludge composting. Bioresour Technol 210:160–166. doi:10.1016/j.biortech.2016.01.094 26852272

